# Theoretical Discussion of Applicability and a Practical Example of Using Statistical Second-Generation Techniques to Analyze Causal Relationships in Animal Experiments

**DOI:** 10.3390/ani16010124

**Published:** 2026-01-01

**Authors:** Becker Katrin

**Affiliations:** 1 Faculty of Communication and Business, IST University of Applied Sciences, 40233 Düsseldorf, Germany; katrin.becker@uni-duesseldorf.de; 2 Institute for Neurophysiology, Faculty of Medicine, University Hospital Cologne, University of Cologne, 50937 Cologne, Germany

**Keywords:** 3R, statistical method, pathway modulation, R

## Abstract

In animal experiments, causal relationships in physiological or disease processes are investigated by using interventions. Applying second-generation statistical methods could be used to identify important links in life processes. This article as a first step describes how second-generation statistical methods that are often used in social sciences are currently applied in veterinary medicine, including a single-animal experimental study, or an ecotoxicity study in fish. It explains how second-generation statistical methods allow flexible modeling to simultaneously calculate causal relationships between constructs in several layers. It continues with a discussion on how theoretical concepts from this statistical approach could be transferred to experimental or medical data. As an applied example, an investigation on a data set analyzed with a second-generation method is presented, showing how this allows us to calculate relationships between variables within a complex theoretical model. Limitations of the use of second-generation statistical methods as strict requirements on the data sets are overcome by technical developments; however, causality cannot be established by statistically testing hypothesized causal structures. Using second-generation statistical methods in the future might promote obtaining more data from one animal and thereby potentially even reducing animals in line with the 3R principle.

## Simple Summary

In animal experiments, causal relationships in life processes are, for example, investigated with pharmacological interventions, surgeries, or genetic manipulations. With the aim of obtaining more data from one animal and thereby potentially even reducing animals in line with the 3R principle, collecting multimodal data from single animals could be used to statistically identify important links in life processes. We therefore suggest and give examples for the use of second-generation statistical models such as structural equation modeling (SEM) or the partial least squares method (PLS). Second-generation statistical methods are often used in social sciences. Applications in veterinary medicine are found in breeding management of farm animals, epidemiology, veterinary care, and an ecotoxicity study in fish. Second-generation statistical methods allow flexible modeling to simultaneously calculate causal relationships between constructs in several layers. Observed variables serve the definition (formative model) or measurement (reflective model) of the latent variables, which present processes that cannot be directly observed. The factor-based common factor model investigates if empirical measurements of a concept agree with the suggested theoretical nature of a concept. Its alternative is composite-based and uses constructed artifacts. The theoretical question arising from this is whether factors or composites exist in medicine.

## 1. Introduction

According to the German animal protection act [1] §1, no person may, without good cause, inflict pain, suffering, injury or lasting harm on any animal. Death of an animal is one form of harm, and, accordingly, §17 of the animal protection act states that killing an animal without reasonable cause is a criminal offense. Killing of animals in animal experiments (§7 of the animal protection act) in accordance with EU directive 2010/63 [2] must adhere to the 3R principle (replace, reduce, refine) [3], of which the second R stands for reduce, that is, limiting the number of animals used to the required minimum. Upon planning an animal experiment, one must provide a power calculation to demonstrate that the animal proposal does only comprise the minimum possible number of animals.

To investigate disease processes in animals, to date, interventions such as pharmacological treatments or surgeries, or genetic manipulations, are necessary which, for example, inhibit or activate signal transduction pathways to find out which effect this has on a pathophysiological process. For this purpose, treatment and untreated control groups are required or genetically manipulated and wildtype control animals. Additionally, a certain group size is necessary to obtain statistically significant results.

Against this background, it is suggested that complex correlation analyses of several data sets obtained from one single animal by multimodal measurements can be performed using a second-generation statistical method. This would allow one to conclude on the relationships between these data sets. Ideally, by this, it is possible to, for example, find out which signal transduction pathways or metabolic pathways play a role in the development or course of a disease, or its therapy.

In the future, by using second-generation statistical methods, reduction in animal numbers by minimizing the number of intervention groups can be obtained on one hand by using pilot studies with small numbers of animals, in which multimodal analyses with subsequent complex statistical analyses are performed to identify important paths which should be further investigated. On the other hand, more information can be gained from a single animal which might serve to reduce intervention groups, as interconnections which before had to be modulated pharmacologically or by surgeries can be calculated from the underlying correlation analyses. Complex statistical analyses of animal experimental data by second-generation statistical methods therefore are a potentially important contribution to “reduce” in line with the 3R principle.

Second-generation statistical methods are often used in the social sciences, as in marketing or psychology. In the veterinary medicine field, they are used in planning the breeding of farm animals [4], epidemiology, and veterinary care [5]. Only one animal experimental study exists, an ecotoxicology study in fish, in which the second-generation method of structural equation modeling (SEM) is used to determine which key events cause the death of animals [6].

That study is an interesting example on the use of PSEM (piecewise structural equation modeling), which is an advanced form of SEM, on experimental animal data and on how animal numbers in toxicology studies can be saved by using complex statistical analyses. The authors investigate an adverse outcome pathway network consisting of several linear networks. From their data, they calculated which of the linear networks contribute most to adverse outcomes, and if any upstream key events could predict the adverse outcomes. Their adverse outcome pathways consist of a cascade of causal toxicology events, starting with a molecular-initiating event that extends over multiple key events—the events that occur in complex biological organisms—and ending with an adverse outcome. The study yielded reliable complex adverse outcome pathway networks despite the small number of experimental data and showed that it was possible to identify the most important adverse outcome pathways, as well as the predictive potential of key events. Most results were similar between PSEM and the Bayesian approach also used in this study, which in contrast to PSEM, did not have sufficient sensitivity. The authors suggest that using complex statistical methods serves to reduce animal tests, as the possibility to conclude on causalities between events in complex networks allows us to obtain more information from new analysis methods like in vitro high-throughput screening or high-content omics [6].

Second-generation statistical methods might also be applied in the analysis of experiments on laboratory animals, to collect more information from one animal. This would allow us to reduce the number of intervention groups, as necessary information can already be calculated, instead of by analyzing differences between treated and control animals. In addition, this approach allows a more targeted planning of interventions, if only the most promising interventions are performed by prior identification of important links in pathophysiological processes.

Aim of this article is to, after an introduction to second-generation statistical methods, perform a theoretical transfer of this statistical technique to animal experiments by discussing how theoretical concepts like composites and factors can be applied on experimental data and disease processes. The second step towards application is to give an example for a second-generation analysis on a small data set using PLS and to explain how this helps to reduce animal numbers.

## 2. Second-Generation Statistical Models and Their Potential Applications in the Analysis of Animal Experiments

For the calculation of second-generation statistical analyses, R [7] (version 4.3.3) is used, which is an open-source software background for statistical applications, which by an active community is continuously improved. This is possible by development of libraries by users, which allow multiple applications. Upon upcoming problems, it is possible to obtain support, and identified flaws are corrected timely. One problem for inexperienced users is that they must learn a programming language, as the software has no simple user-friendly point-and-click surface.

Statistical methods can be subdivided into first- and second-generation techniques [8]. First-generation techniques are, for example, mean difference tests such as *t*-tests and ANOVA, as well as correlation and regression analyses. These methods provide limited opportunities to work with models. They cannot directly investigate the causal relationships between three or more constructs but must reduce these to the calculation of relationships between pairs. That implies that they investigate the plausibility of a single cause–effect relationship and must proceed stepwise for more complex relationships. For the investigation of paths, path analyses can be used.

**Figure 1:**
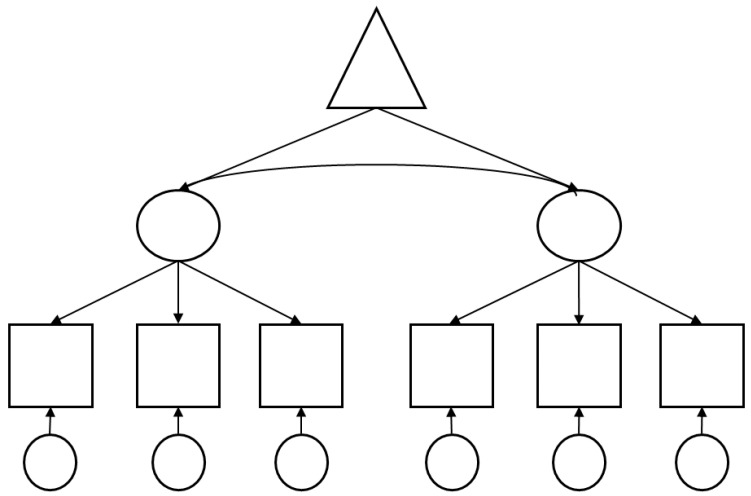
Schematic of a model for a second-generation statistical method calculation. It is possible to perform a calculation of causal relationships up to several levels. Triangle: exogenous latent construct; oval: endogenous latent construct; square: observed variable; circle: error term.

Second-generation techniques in contrast, allow comprehensive and flexible modeling for the calculation of causal relationships. One of these techniques is covariance-based structural equation modeling (CB-SEM), which in recent decades, has been widely applied. Other techniques are partial least squares (PLS) path modeling [9], or newer techniques, which try to circumvent some of the problems of the older techniques, such as that of strict requirements on the data sets [10]. Second-generation techniques can investigate a complex collection of hypotheses of a causal theory simultaneously (please see Figure 1), making much less mistakes than first-generation techniques; therefore, complex models must be preferred before first-generation methods [11].

Covariance-based structural equation models [12] were especially widely applied in the social sciences in recent years. Requirements on the data are, for example, a large sample size and normal distribution. In addition, the model must be specified correctly [9], which includes the scientist suggesting the correct causal direction (temporal priority) and the absence of confounders [8]. It is difficult to find data sets which fulfill these requirements [9]. Therefore, as an alternative, the partial least squares technique is often applied, which is supposed to not pose the same strict requirements on the data sets. However, in the literature, there is a vivid discussion regarding this technique, as no scientifically sound explanation is given as to why these requirements are supposed to not apply when using PLS. In addition, upon using PLS, other errors, such as confounders, arise [10]. However, modern PLS-SEM is a well-established technique [13]. Newer applications which develop techniques like covariance-based SEM further tackle the problem of small sample sizes and missing normality distribution, via correction of the Chi^2^ statistics, multivariate transformation, modified testing statistics, robust estimators [10], or confidence intervals with Monte Carlo simulation, and correction for confounders.

Therefore, these techniques should be suited for the statistical analysis of data acquired in animal experiments.

The relationships in second-generation or in simple first-generation analyses are presented as paths. They represent cause–effect relationships, which are investigated in a hypothesis test in which the correlation between two variables is identified. The complex second-generation analyses contain paths for every hypothesis in a theory, which allows a comprehensive testing of multilevel theoretical relationships [8]. These paths can identify both direct and indirect effects. Graphically, paths are often displayed as arrows.

**Figure 2:**
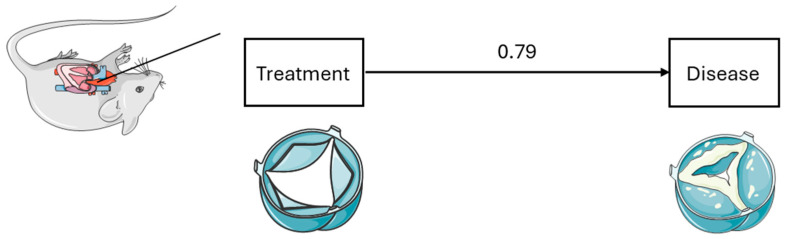
First-generation analysis. Servier Medical Art, CC BY 4.0, https://creativecommons.org/licenses/by/4.0/ (accessed on 5 September 2024).

The next figure (Figure 2) is an example of a first-generation analysis. In this case, an analysis, for example, of the effect of surgical intervention on the development of a disease can be assessed, which is a simple cause–effect relationship.

**Figure 3:**
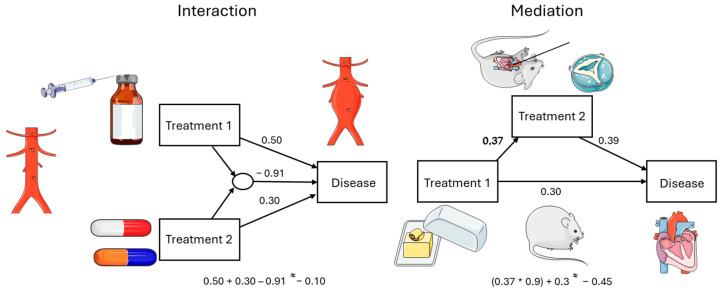
Interaction, mediation—path analyses. Servier Medical Art, CC BY 4.0, https://creativecommons.org/licenses/by/4.0/ (accessed on 5 September 2024).

The example in Figure 3 is a more complex path analysis. There is no simple cause–effect relationship, but moderators and mediators are involved in the relationship between the independent and the dependent variable. On the left, an interaction is displayed, in which the moderator experiences the effect of the combination of two different treatments being different from the sum of the isolated effects of the two treatments. For example, if the isolated treatments increase a symptom, their combination could result in a reduction in the symptom or its over proportional increase.

In case of a mediation, as displayed on the right side of Figure 3, treatment does not only have a direct effect, but also, via the effect of a mediator, has an indirect effect on the development of a symptom. An example from an experimental animal study is a high-fat diet, which in addition to causing diabetes mellitus, also negatively affects heart function, via effects on the aortic valve itself which influence the sequelae of an aortic valve surgery. By aggravating the aortic valve stenosis, it further reduces cardiac performance.

**Figure 4:**
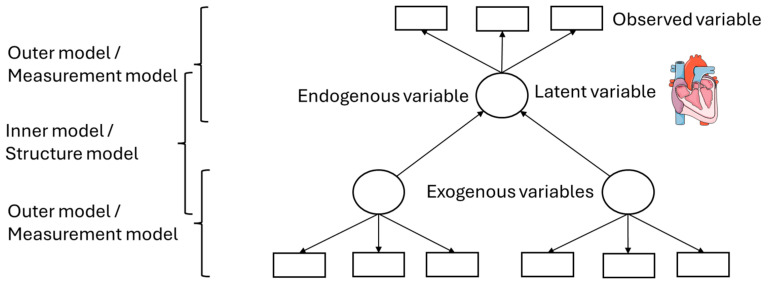
Simple second-generation analysis model. Servier Medical Art, CC BY 4.0, https://creativecommons.org/licenses/by/4.0/ (accessed on 5 September 2024). Squares: observed variables; circles: latent variables.

Figure 4 is an example of a simple second-generation statistical analysis. The squares represent observed variables or indicators; the variables can be measured. The circles represent the latent variables, that is, theoretical assumptions about the underlying mechanisms, of which the effect can be measured via the observed variables. Endogenous variables are latent variables, which depend on the other latent variables—represented by arrow heads pointing at those latent variables—while exogenous variables are not affected by other latent variables, that is, they are independent [9]. This even becomes more complex as single latent variables can technically act as independent and dependent variables for different parts of the model. The model, because of these components, can be subdivided into an inner and an outer model. The inner model or structure model describes the relationships between the independent and dependent latent variables, while the outer or measurement model describes the relationships between the latent variables and their observed variables [9].

Measurement models can be differentiated into reflective and formative. The reflective models assume that the indicators, that is, the observed variables, are an effect of the latent variables. In the graphical visualization, this is represented by arrows from the latent variables to the indicators. Changes in the latent construct cause changes in the indicators. Regularly, the common factor model is used for reflective models [8,14,15].

The formative model in contrast assumes that the indicators define the latent construct, that is, that the construct is a function of its indicators. The arrows point from the observed to the latent variables. For operationalization of these concepts, composite models are usually used [8,14,15].

**Figure 5:**
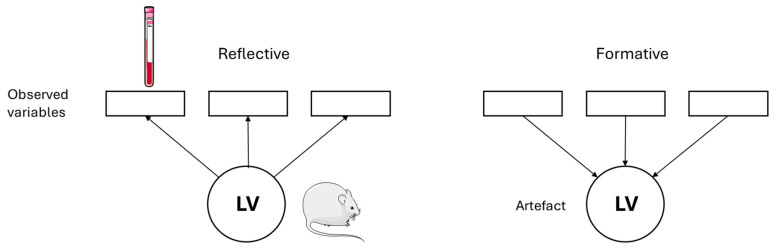
Reflective and formative measurement model. Servier Medical Art, CC BY 4.0, https://creativecommons.org/licenses/by/4.0/ (accessed on 5 September 2024). Squares: observed variables; circles: latent variables.

Animal experiments, in which the effects of treatments on the development of a disease are described, are suggested to be best described by reflective models as an event in the organism that is measured by different markers, such as blood parameters, which are altered if the process in the organism changes (please see Figure 5).

## 3. Factor Analysis, Composites, and Their Potential Application to Animal Experimental Data

This section deals with the possibility of transferring the theoretical models behind the second-generation statistical techniques to animal experimental data. They are the basis for setting up the calculations in R, as, for example, the paths have different directions in different models.

Jöreskog [16] invented the common factor model as a factor-based measurement model, which investigates if empirical measurements of a concept are in line with the assumed theoretical nature of this concept [17]. In that model, researchers aim to understand the behavior of non-observable, conceptual variables, which from a scientific perspective represents real units and possessing direct and indirect causal consequences in the real world. From an empirical perspective, they are terms for observable empirical regularities, while only the observable phenomena are considered as real. Operationalists in contrast consider them as applications of a defined quantitative method for data collected after a specific protocol [14]. The common factor model assumes that the observed indicators are a manifestation of the underlying concept, which is assumed as their common cause [17]. In recent years, the operationalization of the theoretical concept and of the common factor has increasingly merged, so that the terms are hardly distinguishable [17].

Wold [18] invented a composite-based alternative to this approach. The composites are used for the investigation of constructed artifacts and abstract concepts, which serve a certain cause, and of which the indicators do not necessarily have a common cause [15,17]. They initially served the purpose of dimension reduction; therefore, they were conceptualized such that they as efficiently as possible captured the most important characteristics of the data [17].

For a long time, in covariance structure analysis, abstract concepts were operationalized by latent variables (LVs) and called LV modeling [19]. The indicators in these models are the cause of the LVs, supplemented by an error term, which represents missing causes. Each change in a formative LV is mediated by a change in at least one indicator or in the error term [20].

In the initial composite model, the constructed concept is displayed as an emergent variable (named composite construct, aggregated construct, or formative construct). The emergent variables are defined by their indicators, that is, they are a linear combination or summary of observed variables, instead of causing them, as in the common factor model [15,19]. The emergent variables can be embedded in structure models, as LVs are in SEM. They are estimated by different methods amongst others by PLS or via main component analysis, linear discrimination analysis (LDA), or generalized canonical correlation analysis (GCCA) [19].

Rigdon [21] developed these concepts further: He suggested interpreting factors (common factors) or composites, which are the statistical models, as approaches for conceptual variables and theoretical models which cannot be observed. In this way, he explains the relationship between the concepts and the associated observed variables [14].

For the models which are used in animal research, the question arises, under the assumption of the measurement model applied, whether the aim is to obtain results which reflect the real situation as good as possible.

In line with the theory of Jöreskog [16], factors exist in reality but cannot be measured directly. An example from economics is the attitude towards a brand. Composites in line with the theory of Wold [18], in contrast, are artifacts, which are created by humans, and therefore do not exist in nature. However, they can be meaningful, for example, in economy, in the form of stock indices as the NASDAQ composite. In the theory of Henseler [17], in this relationship, the term “therapy” is used.

Therefore, some questions arise:

(1) Do factors exist in (veterinary) medicine? If so, a composite cannot be a solution, even if the causes of the factor are of interest. In this case, the MIMIC (multiple indicators, multiple causes) model of Jöreskog [22] would be of interest.
(2) Do composites exist in (veterinary) medicine? This is not known; however, in this relation, the term “therapy” from the theory of Henseler [17] is probably accurate.

These considerations are probably philosophical science.

If it is assumed that the disease of interest is a factor, and the symptoms are modeled as a consequence of the disease, this is a reflective measurement model. If it is of interest which treatment has which influence on the factor (the disease), this is equivalent to the MIMIC model of Jöreskog [22] with multiple indicators (symptoms) and multiple causes (treatments).

## 4. Practical Example of a Second-Generation Analysis Using the Partial Least Squares Method

### 4.1. Theoretical Background of PLS

The partial least squares method (PLS) is used, for example, in linguistics and educational sciences, as well as marketing and information systems [11]. This technique was first presented in 1982 by Wold. It was initially developed as it requires less computing capacity than classical SEM techniques, which, compared to current standards, was easier to handle for the computers with lower computation capacity. Later, Ringle et al. [13] developed the computer software SmartPLS (latest version 4.1.1.6).

While covariance-based SEM (CB-SEM) works with variance–covariance matrices, from which as constructs (i.e., latent variables) factors can be defined and model theoretical covariances can be derived (i.e., covariances between the indicator are the product of the causative factors; factor theoretical approach), PLS works with raw data. Hereby, PLS represents latent variables as components (composites), that is, as linear combinations of the indicators/observed variables (component-based method). In this way, via estimates, predictions are made over the connections between the variables.

The results of PLS and CB-SEM cannot be directly compared, as both methods use different algorithms for the estimation of model parameters [8,11].

The main causes for using PLS are small sample sizes, non-normally distributed data, complexity of the model (many constructs and indicators, reflective and formative measurement models, mediation and moderation effects, constructs of higher-order, non-linear relationships), theory development, and explorative work, as well as the predictive focus of the study [23]. However, in an academic debate, the scientific foundation of these considerations is put into question. Similarly, options for quality control and model comparisons are limited.

A PLS analysis is composed of two steps: First, by using the main axis or main component analysis, observed variables are combined to latent variables graded by scores (components). Then, by using significance tests of the null hypothesis, separate regression analyses are performed for the composites, by investigating the relationship of a regression coefficient and its standard error of the bootstrap with Student’s *t*-test [10].

### 4.2. Practical Example

**Figure 6:**
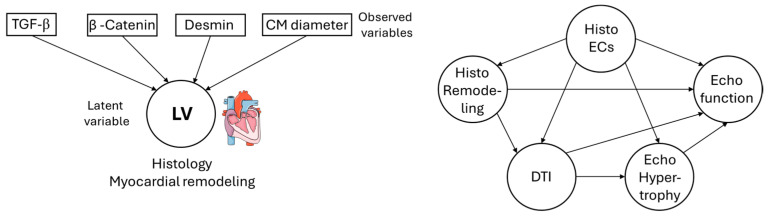
Pathway analysis with partial least squares (PLS) using the PLSPM-package [24] in R 4.3.3 and R studio 28.2.6. Presented here are the direct effects between the observed variables (histology markers for cardiac remodeling) on their respective latent variable (histology remodeling) in the outer model (**left**) and between latent variables in the inner model (**right**). CM: cardiomyocytes; DTI: diffusion tensor imaging; EC: endothelial cell; LV: latent variable. Adapted from [26]. Servier Medical Art, CC BY 4.0, https://creativecommons.org/licenses/by/4.0/ (accessed on 5 September 2024).

As a practical example on the application of a second-generation statistical analysis on an experimental animal data set, an analysis with PLS [24] was performed on the data obtained from n = 3 mice which underwent wire-injury aortic valve stenosis surgery [25]. From these animals, echocardiography was performed over four weeks after surgery, and after four weeks of histology staining and diffusion tensor imaging (DTI) with MRI on explanted hearts to quantify amongst others myocardial fibrosis. Figure 6 gives an example of how the results of such an analysis look like. It shows the direct effect of observed variables on their latent variable in the outer model (left) and direct effects between latent variables in the inner model (right). With values above 0.5 or 0.7, or below the respective negative values, the respective variables have high-positive or negative contribution to their respective latent variables.

This information on the importance of single markers to a larger process and on the effects of different latent variables within the process on each other can be obtained by analyzing the combination of data which gives a more complex picture of the processes than, for example, mean differences analyses or a regression analysis. In the case that a strong impact of certain latent variables on each other is found in the inner model, in the analysis of the outer model, markers which have an impact on that latent variable can be identified. In subsequent analyses, the researcher can then focus on the most important markers identified in this prior analysis, for example, by using knock-out animals for the marker with the most important impact on the process of interest, instead of having to do analyses on knock-out animals for all markers.

### 4.3. Criticism on PLS

PLS is a technique which has been applied for 50 years. In recent years, this method has come into fashion, since it was suggested that the strict requirements which are posed on the data sets when using the CB-SEM technique do not apply for PLS [11]. However, the fact that the software which uses PLS even produces results if those requirements are not met does not imply that those results are valid. A scientific explanation on the application of the PLS causing the requirements to not apply has not been given so far [10].

The PLS has additional drawbacks. On the one hand, latent variables per definition cannot contain scores. On the other hand, the application of scores produces bias.

Also, the explanation that CB-SEM cannot be applied for data sets which do not meet the strict requirements, like large sample numbers and normal distribution, in recent years has been met by several developments of the much more solid CB-SEM method. Rönkkö et al. [27] state corrections of the Chi^2^ statistic for the adaptation of the model size effect (that is, small N:p ratio) [28,29], as well as many techniques for dealing with non-normally distributed data, such as multivariate transformation [29], modified test statistics [30], and robust estimators [31].

There is strong criticism on PLS in a specific academic debate which states that PLS is not an SEM method, even if, in the literature, it is often referred to as one. Rather, PLS is an indicator weighting system for the creation of combined variables [10]. Both steps of the PLS can be better solved by the structural after measurements method (SAM), in which, subsequently and not simultaneously as with SEM, the measurement and structure model are investigated. This method has the advantage of being less sensitive in case of local misspecification of the model, and being less susceptible to convergence problems in case of small sample sizes; it also shows smaller finite sample biases in correctly specified models [32].

In addition, PLS is criticized as having no coherent theoretical foundation for estimation and causal conclusion. Different from classical SEM, which is based on a uniform statistical theory for simultaneous estimation of all measurement parameters, the PLS approach is based on an ad hoc collection of statistical procedures, which has not been formally analyzed [27].

Rönkkö et al. [27] state additional problems of PLS in terms of its limitations in global model testing, problems with significance testing of path coefficients, extremely high false-positive rates upon application of empirical confidence intervals in combination with a new sign-change correction for path coefficients, conceptual and statistical problems for formative measurements, and limitations regarding the ability to investigate how well a given theoretical model represents the available data (for example, lack of a well-established overidentification test).

There is also criticism of PLS. The critics would use incorrect reasons as to why PLS cannot be applied by assigning favorable properties to factor-based SEM, which this method does not possess. The faulty arguments against PLS were as follows:

- It creates biased parameter estimates—the studies which are supposed to show this would use mis-specified PLS models and other flaws in the design, and the bias would be reduced with increasing sample sizes.
- PLS is no LV method: LV, as well as the term construct, would be terms with different meanings, and both would be conceptual variables in a theoretical model, which influences the behavior of another variable or of a common factor; to deny this, the PLS would be playing upon words.
- There is no common fit test for this model. Also, the Chi^2^ test of the factor-based method could not ensure that the factors are valid approximations of certain conceptual variables.
- The measurement errors are not displayed; similarly, none of these factor-based approaches would protect against measurement errors, and there would be no hint that the factor-based method is better than the composite-based method.
- Modern PLS-SEM (as implemented in SmartPLS [13]) is a well-established variance-based SEM method for prediction and theory development, especially for complex models and non-normal data.

In addition, it cannot generally be assumed from common factor approaches that they, regarding the existence or nature of conceptual variables, display larger significance than composite-based approaches [14].

The results, which are created with PLS, therefore must be treated with caution. As an alternative, classical SEM techniques could be used. In the case of small sample sizes, the applicability of SEM techniques must be examined to find appropriate measurement parameters, with which reliable results can be generated. This is the aim of an ongoing study [33].

## 5. Discussion

This article gives an introduction to how second-generation statistical methods can be used on experimental animal data sets. After introducing these methods, it discusses how theoretical concepts of second-generation statistical methods can be transferred to experimental data and disease processes, as a necessary basis for setting up a calculation in R. Lastly, an example of how to calculate a complex statistical analysis on an experimental data set is given, due to small n-numbers using PLS. The next section of this article mainly focusses on the criticism of PLS and on alternatives to this technique, as, to date, this second-generation technique is widely used on small data sets and other data which do not meet the strict requirements necessary to calculate classical SEM analyses, like what is often found in animal experimental data. The appropriateness of this, however, is put into question by the community.

Second-generation statistical methods are regularly applied in social sciences; however, they have not been regularly used in animal experiments. They are suited for modeling complex causal relationships, in which they are superior to simpler methods, investigating simple cause–effect relationships between variables; therefore, they must work step by step. By developing new techniques, it might be possible to work with data sets which only comprise small sample numbers and which are not normally distributed. This is often the case with animal experimental results and is even asked for due to the ethical necessity to reduce animal numbers.

As newer techniques that are suited for the analysis of causal relationships in small sample sizes exist [27,28,29], application of second-generation statistical methods due to several reasons is highly relevant for animal experimental research. On one hand, from several data sets acquired in one animal, causal relationships can be calculated, without having to use additional intervention groups or genetically modified animals. This, besides the animals included in the analysis, also comprises surplus animals from breeding. Besides reduction, this serves as refinement by limiting the burden the experimental animals must endure due to experimental manipulations. At the least, these advanced statistical calculations allow a more straightforward experimental approach, by promising signal transduction pathways can be identified by statistical calculation and the subsequent experiments limited to these pathways.

### Limitations

An important limitation of second-generation statistical methods is that they test hypothesized causal models derived from theory. However, they cannot calculate causal relationships from observed data alone. Instead, randomized interventions (such as pharmacological or surgical interventions or genetic manipulations) are designed to specifically infer causality. Complex statistical analysis can be applied to data from within an experimental group to model the complex, multi-factorial relationships between measured variables like food and blood parameters, as well as functional values like heart function in echocardiography but cannot replace the comparison between experimental groups against control groups. However, in times in which authorities are ever more restricting access to animal numbers and intervention groups, besides the ethical aspect, restricting the groups in which interventions are performed to a minimum by obtaining as much information on the processes of interest by complex statistical analyses is of great importance.

## 6. Conclusions

The application of second-generation statistical methods is potentially an important contribution to reduce and refine, in line with the 3 R principle. The development of techniques which produce reliable results from small sample sizes and non-normally distributed data sets is therefore an important task.

## Data Availability

No new data were created or analyzed in this study. Data sharing is not applicable to this article.

## Abbreviations

The following abbreviations are used in this manuscript:

3R	3R principle (replace, reduce, refine)
CB-SEM	Covariance-based structural equation modeling
CM	Cardiomyocyte
DTI	Diffusion tensor imaging
EC	Endothelial cell
GCCA	Generalized canonical correlation analysis
LDA	Linear discrimination analysis
LV	Latent variable
PLS	Partial least squares
PSEM	Piecewise structural equation modeling
